# The Nakata index and McGoon ratio: correlation with the severity of pulmonary regurgitation after the repair of paediatric tetralogy of Fallot

**DOI:** 10.1186/s43044-023-00423-9

**Published:** 2023-11-29

**Authors:** Reza Abbaszadeh, Raheleh Askari-Moghadam, Maryam Moradian, Hojat Mortazaeian, Mohammad Reza Safaei Qomi, Negar Omidi, Yasaman Khalili, Tahmineh Tahouri

**Affiliations:** 1 Rajaei Cardiovascular Medical and Research Center, Iran University of Medical Sciences, Tehran, Iran; 2 Department of Paediatrics, Arak University of Medical Science, Arak, Iran; 3 Cardiac primary Prevention Research Center, Cardiovascular Disease Research Institute, Tehran Heart Center, Tehran University of Medical Sciences, Tehran, Iran; 4 Shahid Modarres Educational Hospital, Faculty of Medicine, Shahid Beheshti University of Medical Sciences, Tehran, Iran

**Keywords:** Fallots tetralogy, Pulmonary arteries, Pulmonary regurgitation, Congenital heart defect, Cardiac surgery

## Abstract

**Background:**

Pulmonary regurgitation is the most common complication after the complete repair of tetralogy of Fallot, and severe pulmonary regurgitation after surgery requires pulmonary valve replacement. In this retrospective observational, cross-sectional study, we included a total of 56 children aged 6 years or younger who underwent complete repair of TOF at Shahid Rajaei Cardiovascular Medical and Research Center in Tehran, Iran. Preoperative dual-source computed tomography was used to measure the McGoon ratio and Nakata index. The patients were divided into two groups based on the severity of postoperative pulmonary regurgitation, as estimated by trans-thoracic echocardiography: the severe pulmonary regurgitation group and the non-severe pulmonary regurgitation group. The McGoon ratio and Nakata index were then compared between the two groups.

**Results:**

When comparing the two groups, we found that the corrected right pulmonary artery diameter, main pulmonary artery diameter, and McGoon ratio in the non-severe pulmonary regurgitation group were higher than in the severe pulmonary regurgitation group. However, none of these differences were statistically significant. Additionally, other variables, including the corrected left pulmonary artery diameter and Nakata index, showed higher measurements in children with severe pulmonary regurgitation, but again, the differences were not statistically significant.

**Conclusions:**

This study indicates that pulmonary arteries diameter, Nakata index, and McGoon ratio were not significantly correlated with the severity of pulmonary regurgitation after the complete repair of tetralogy of Fallot.

## Background

Tetralogy of Fallot (TOF) is the most common cyanotic congenital heart disease, accounting for 10% of all congenital heart diseases. TOF is a conotruncal anomaly that includes ventricular septal defect, aorta overriding, right ventricular outflow tract obstruction, and right ventricular hypertrophy [1]. The presentation of TOF varies depending on the severity of the right ventricular outflow tract obstruction, with greater severity leading to earlier appearance of cyanosis [2].

Timely treatment is crucial, as untreated TOF can have a mortality rate of 95% by the age of 40. The surgical approach for TOF treatment has evolved over the years, with the systemic to pulmonary artery shunt being the first method introduced by Blalock, Taussig, and Thomas in 1944, followed by the total correction surgery method by Lillehei et al*.* in 1955 [3, 4]. The ideal TOF repair aims to close the ventricular septal defect and relieve the right ventricular outflow tract obstruction [5]. Total correction of TOF (TFTC) in children usually yields favorable outcomes and long-term survival of about 90%, but there can be complications such as pulmonary regurgitation (PR) [6].

PR is the most common complication after TFTC, potentially leading to right ventricular dysfunctions and tachyarrhythmia. Severe PR after surgery may require pulmonary valve replacement, so close post-TFTC evaluation is essential to detect and assess residual hemodynamic lesions, including the severity of PR [7].

While trans-thoracic echocardiography is a routine method for clinical assessment and monitoring of TOF patients, dual-source computed tomography (DSCT) has emerged as a valuable technique to assess the cardiovascular system, particularly pulmonary artery branches in TOF patients [8]. However, limited researches have investigated the correlation between the DSCT findings and the severity of PR after TFTC.

This study aims to examine the correlation of the McGoon ration and the Nakata index with PR severity and right ventricular function after TFTC. The McGoon ratio, obtained by dividing the sum of the sizes of the widest portion of the right and left branches of the pulmonary artery by the diameter of the descending aorta at the level of the diaphragm, provides valuable information for pulmonary arteries sizes assessment. The McGoon ratio can be calculated through cardiac catheterization, cardiac MRI, and CT scan [9]. Similarly, the Nakata index, obtained from the sum of the areas of the right and left branches of the pulmonary artery divided by the body surface area, is a relevant parameter [10]. One-stage total correction of TOF can be considered in patients with a McGoon ratio above 1.2 and a Nakata index above 150 mm^2^/m^2^ [8]. The aim of this study was to investigate whether preoperative DSCT findings can predict the severity of pulmonary regurgitation after complete TOF repair.

## Methods

This retrospective observational, cross-sectional study utilized data from the Shahid Rajaei Cardiovascular Medical and Research Center (RCMRC), a tertiary care hospital for cardiovascular patients in Tehran, Iran. Ethical approval was obtained from the RCMRC’s ethical committee (IR.IUMS.FMD.REC.1400.412) on October 9, 2021, and informed consent was obtained from the parents of the participating children.

This study included children aged 6 years or younger who underwent complete repair of TOF between March 2018 and March 2020. All children underwent DSCT before the surgery and trans-thoracic echocardiography one month after the surgery. Exclusion criteria were age > 6 years old at the time of surgery, missing or incomplete clinical chart records, critical or life-threatening conditions, and pre-existing presence of pulmonary regurgitation.

### Data collection

Data collection encompassed demographic information, BMI, BSA, history of shunt surgery, and various trans-thoracic echocardiography parameters and DSCT findings. The BMI and BSA were calculated using the following formulas:

$${\text{BSA }}\left( {{\text{m2}}} \right){\mkern 1mu} = {\mkern 1mu} \sqrt {\left( {\left[ {{\text{Height }}\left( {{\text{cm}}} \right){\mkern 1mu} \times {\text{ Weight }}\left( {{\text{kg}}} \right)} \right]{\text{ }}/3600} \right)} {\mkern 1mu}$$

$${\text{BMI }} = {\text{ weight }}\left( {{\text{kg}}} \right) \, /{\text{ height }}\left( {\text{m}} \right) \, \times {\text{ height }}\left( {\text{m}} \right)$$

A cardio-imaging fellowship measured left pulmonary artery (LPA) and right pulmonary artery diameters (RPA). Multiplanar reformation (MPR) was used to analyze DSCT. The McGoon ratio was calculated by the sum of LPA and RPA diameters divided by the aorta diameter at the diaphragm level. The Nakata index was calculated by the sum of LPA, RPA, and collateral vessels’ cross-sectional areas divided by body surface areas. Postoperative echocardiography was conducted by a single experienced pediatric cardiologist. The quantification of the pulmonary regurgitation was done using color Doppler flow imaging of the central pulmonary arteries from the parasternal short axis view. Both the origin of the diastolic backflow and the width of the regurgitant jet were measured. All the echocardiographic images were obtained by a GE vivid S6 (General Electric Medical Systems, Chicago, Illinois, USA).

### Data analysis

Patients were divided into two groups. The severe pulmonary regurgitation group includes those with severe PR after surgery, and the non-severe pulmonary regurgitation group includes those with mild, moderate, or no PR after surgery. DSCT measures and the number of shunt operations were compared between the groups, and the Nakata index and McGoon ratio were also assessed, taking shunt history and z-scores in pulmonary arteries into account.

Data analysis was performed using SPSS software for Windows (version 26, SPSS Inc., Chicago, IL, USA). The one-sample Kolmogorov–Smirnov test was used for the normality test, and differences in variables between different data groups were assessed using the Mann–Whitney U test and Kruskal–Wallis test, with statistical significance set at *p*-value ≤ 0.05.

## Results

### Patient’s characteristics

During a two-year period, 94 children underwent surgical repair of TOF. After excluding 38 participants for specific reasons (such as age at surgery, incomplete or missing DSCT reports, and critical condition), a total of 56 children were included in the study. The mean age at the time of surgery was 23.2 ± 14.4 months, ranging from 6 to 71 months. Among the participants, 29 (51.7%) were female, and 27 (48.2%) were male. Nine children had a history of shunt operation (see Table 1).

**Table 1:** Participants' characteristics

Demographics	*N* (%)
*Age*	
Mean (SD), months	23.2 ± 14.4
Range, months	6–71
*Sex*	
Female	29 (51.8%)
Male	27 (48.2%)
*Shunt operation history*	
Yes	9 (16.1%)
No	47 (83.9%)
*Shunt type*	
Right	4 (7.1%)
Left	4 (7.1%)
Right + Central	1 (1.8%)

SD: Standard deviation

### Relationship between DSCT findings and the severity of pulmonary regurgitation after total correction of tetralogy of Fallot

The participants were divided into two groups based on the severity of pulmonary regurgitation (PR). Severe pulmonary regurgitation was defined as originating the diastolic reversal flow from the distal pulmonary arteries with the width of the color jet to right ventricular outflow tract > 0.5. Additionally, a pressure half time of < 100 ms, as measured using continuous wave Doppler, was considered indicative of severe pulmonary regurgitation. Patients who did not meet these criteria were placed in the non-severe PR group. The severe PR group consisted of 16 children with severe PR, while the non-severe PR group included 40 children with mild, moderate, or no PR. Right pulmonary artery, left pulmonary artery, main pulmonary artery (MPA) diameters and areas, McGoon ratio, and Nakata index were compared between the two groups.

Comparison between the two groups showed that the corrected right pulmonary artery (RPA) diameter, RPA area, main pulmonary artery (MPA) diameter, MPA area, and McGoon ratio were higher in the non-severe PR group than in the severe PR group. However, none of these differences reached statistical significance. Other variables, including corrected left pulmonary artery (LPA) diameter, LPA area, aortic diameter, and Nakata index, exhibited higher measurements in children with severe PR compared to the other group. The differences were also not statistically significant (Table 2).

**Table 2:** Severity of PR based on the pulmonary arteries diameter, Nakata index, and McGoon ratio

Variable	Severe PR (*n* = 16)	Non-severe PR (*n* = 40)	*P*- value
LPA diameter (mm)	10.0 (7.9–13.4)	9.8 (8.6–12.5)	0.9
RPA diameter (mm)	8.3 (6.8–11.1)	8.8 (7.4–10.07)	0.8
AO diameter at the diaphragm level (mm)	8.3 (7.1–9.3)	8.2 (7.5–9.05)	0.7
LPA area (mm2)	85 (51.7–136.5)	74 (52.2–118)	0.5
RPA area (mm2)	53 (29 -80.2)	64 (44.5–80.5)	0.4
MPA (mm2)	74.6 (45.8–104.7)	86.9 (60–119.5)	0.2
MPA area (mm2)	13.2 (6.7–17.2)	14.2 (9.4–16.9)	0.6
Nakata index	319.5 (222.1–400)	284.1 (222.6–368.6)	0.8
McGoon ratio	2.2 (1.9–2.7)	2.3 (2.0–2.7)	0.8

*Data given as mean IQR*

*PR: Pulmonary regurgitation, LPA: Left pulmonary artery, RPA: Right pulmonary artery, AO: Aorta, MPA: Main pulmonary artery*

### Shunt operation history

The history of shunt operation was compared between the two groups, and no statistically significant differences were found (Table 3).

**Table 3:** Comparison between the severe PR & non-severe PR groups based on the shunt surgery history

Shunt	Severe PR (*n* = 16)	Non-severe PR (*n* = 40)	Total	*P*-value
No shunt	12 (21.4%)	35 (62.5%)	47 (83.9%)	> 0.05
Right	1 (1.8%)	3 (5.4%)	4 (7.1%)	
Left	3 (5.4%)	1 (1.8%)	4 (7.1%)	
Right + Central	0	1 (1.8%)	1 (1.8%)	
Total	16 (28.6%)	40 (71.4%)	56 (100.0%)	

The Nakata index was significantly lower in children with a history of shunt operation compared to those without (*p* = 0.05). However, the McGoon ratio did not show a significant difference between the two groups (Table 4).

**Table 4:** Nakata index and McGoon ratio based on the systemic to pulmonary shunt surgery history

	History of shunt operation	Median (range)	*P*-value
Nakata index	No shunt (*n* = 47)	302.5 (242.6–389.8)	0.05*
	Shunt (*n* = 9)	227.4 (198.0–310.9)	
McGoon ratio	No shunt (*n* = 47)	2.3 (1.9–2.7)	0.8
	Shunt (*n* = 9)	2.2 (1.9–2.8)	

## Discussion

Pulmonary regurgitation is a common complication after TFTC surgery, often leading to valve replacement. Factors, such as trans-annular patching, right ventriculotomy, peripheral pulmonary stenosis, pulmonary vascular resistance, residual atrial or ventricular septal defects, underlying lung diseases, and residual pulmonary valve abnormalities, can contribute to postoperative PR [7].

The measurement of pulmonary arteries diameter, as a part of the preparation for total TOF correction, is a crucial parameter. In this regard, a McGoon ratio of 2 is considered normal, indicating no restriction in the right and left pulmonary arteries. A ratio above 1.2 is acceptable for postoperative right ventricle systolic pressure in total correction of TOF, while a ratio below 0.8 is inadequate for complete TOF repair. Another method, the Nakata index, relies on an average value of 330mm^2^/m^2^ as normal. A Nakata index > 150 mm^2^/m^2^ is acceptable for complete TOF repair, but < 150 mm^2^/m^2^ indicates a narrow pulmonary artery [11].

However, our study found that pulmonary arteries diameter, Nakata index, and McGoon ratio were not correlated with the severity of post-TFTC pulmonary regurgitation. Other studies have reported varying results, with some showing correlations between PR and certain measurements, but our findings suggest otherwise. For example, Gao et al. found a positive correlation between PR and left pulmonary artery diameter, Nakata index, and McGoon ratio, but these were not significant in our study. They concluded that a Nakata index > 270.05 mm^2^/m^2^, McGoon > 1.63, and LPA diameter > 18.29 mm can predict the risk of postoperative pulmonary regurgitation. They explained that the expanded pulmonary arteries may lead to reduced vascular elasticity and compliance. However, similar to our study, postoperative pulmonary regurgitation had no significant correlation with the diameter of the right pulmonary artery or the main pulmonary artery. Their explanation for this was that most of the blood that returns to the heart from the pulmonary artery due to pulmonary regurgitation is more from the left pulmonary artery than from the right pulmonary artery [8]. Similarly, Kilner et al. reported a correlation between larger pulmonary arteries and higher PR fraction [12]. Apandi et al. also associated a McGoon ratio > 1.8 with severe postoperative PR after tans-annular patch repair [13]. In another study conducted by Hennein et al., it was observed that the McGoon ratio and Nakata index did not exhibit a significant relationship with the freedom from reoperation or death [14]. On the other hand, Yuan et al. also reported that the McGoon ratio < 0.89 and the Nakata index < 0.79 were related to early death [15]. Nevertheless, our study indicates that while Nakata index and McGoon ratio are important criteria for deciding whether to perform one-stage or two-stage surgery in TOF patients, but they do not predict postoperative pulmonary regurgitation. However, due to the lack of similar studies in pediatric TOF patients, further investigations are warranted.

Regarding the history of shunt operation, our study did not find a significant relationship with postoperative PR. However, the Nakata index was lower in patient with shunt history. The complete repair of TOF is recommended between 3 months and 1 year old, and palliative procedures such as systemic to pulmonary shunts are indicated in some patients due to small or hypoplastic pulmonary arteries [16]. Although the overall results of palliation with systemic to pulmonary shunt in TOF patients are favorable, it is possible that the palliation with a modified Blalock–Taussig shunt leads to an increase in the incidence of hypoplasia or distortion of the pulmonary arteries [17] and the hypoplasia or stenosis of the pulmonary arteries can be determinants of the postoperative pulmonary regurgitation [7]. Therefore, theoretically, shunt implantation in TOF patients may be associated with an increased risk of pulmonary regurgitation after surgery. However, this relationship was not found in our study.

In recent years, more studies have investigated the clinical management post-TFTC, particularly on long-term complications and surgical methods. Pulmonary regurgitation is the most common complication after the repair of TOF, and most studies focus on the best time for pulmonary valve replacement. Moving forward, it is crucial to investigate clinical management after TFTC, particularly long-term complications. Understanding parameters that affect the severity of the pulmonary regurgitation after TFTC can aid in determining the timing and type of surgery (one-stage or two-stage). This study suggests that complete repair of TOF can be performed even in patients with small pulmonary artery branches without an elevated risk of severe pulmonary regurgitation.

### Limitations

The statistical significance of data in this study might be affected by the unequal number of participants between the two PR and No PR groups. The inconsistency between the group numbers may also be a reason for the conflicts of the results with existing literature. This study examined the outcomes post-surgery, and there was no available data on long-term follow-up times. Larger sample sizes from different cardiovascular centers and follow-up examinations are recommended for future studies.

## Conclusions

This study showed that the pulmonary arteries diameter, Nakata index, and McGoon ratio were not correlated with the severity of post-TFTC pulmonary regurgitation. The findings also showed that the history of shunt operation may not be related to post-surgery PR. However, the Nakata index was lower in those with a history of systemic to pulmonary shunt implantation operation.

## Data Availability

The authors confirm that the data supporting the finding of this study is available within the article and its supplementary files.

## Abbreviations

TOF: Tetralogy of Fallot
DSCT: Dual-source computed tomography
TFTC: Tetralogy of Fallot total correction
PR: Pulmonary regurgitation
BMI: Body mass index
BSA: Body surface area
LPA: Left pulmonary artery
RPA: Right pulmonary artery
MPA: Main pulmonary artery
